# Aid attitudes in short- and long-term perspectives among Ukrainian migrants and Poles during the Russian war in 2022

**DOI:** 10.3389/fsoc.2023.1084725

**Published:** 2023-02-02

**Authors:** Ivanna Kyliushyk, Agata Jastrzebowska

**Affiliations:** Centre for Research on Social Change and Human Mobility (CRASH), Kozminski University, Warsaw, Poland

**Keywords:** Ukraine, war, long-term, short-term, refugees

## Abstract

The aim of this article is to diagnose aid attitudes among those who potentially need help—help receivers, i. e., Ukrainian refugees—and help givers, i.e., Poles and Ukrainian labor migrants, during the initial stage of the escalation of the Russian war in 2022. By aid attitudes, we mean approaches to both the offering and the acceptance of help during the war in the short and long term. We conducted a small-scale exploratory web survey (Computer-Assisted Web Interview—computer-assisted interview using a website) from March to June 2022, in which the main aims were to explore the needs and offers for both, short- and long-term aid and the gaps between them. Respondents were asked about different types of aid without indicating from whom they wanted to receive this help: the state, NGOs or individuals offering their help. The survey results show discrepancies in what migrants need and what is offered to them in Poland, both from the short and long-term perspectives.

## 1. Introduction

Until the early 2000s, Poland was not a key destination for international migrants. This all changed after Poland joined the European Union (EU), which made it, together with the resulting economic development, an attractive country for labor migrants. Poland also found it necessary to open its borders to such migrants as a result of a strong demographic crisis caused by the emigration of Poles and an aging population. According to forecasts, in 2035, one in four Poles will be retired (Wieńska-Di Carlo and Klaus, 2018). That is why Poland among other EU member state offers the most liberal access to its labor market for foreigners from non-EU countries (primarily from Eastern Partnership countries, that is, Ukraine, Belarus, Moldova, Georgia, Armenia and in addition to that also Russia), who are permitted work in Poland even without any qualifications, and Ukrainian citizens, who are even permitted visa-free travel. Accordingly, at the end of February 2020, there were 2,213,594 foreigners living in Poland, of whom 1,390,978 were Ukrainian citizens (Główny Urzc̨d Statystyczny, 2020).

Despite its openness to labor migrants, Poland has not applied an elaborate migration and integration policy strategy and, moreover, it has not been characterized by an openness toward refugees (Głowiak, 2021). This was expressed in Poland's refusal to accept refugees from Syria, Eritrea and neighboring countries in 2015, by which it also refused to support EU member states in dealing with the consequences of the Syrian crisis, in which Russia played a large role. A similar lack of openness to refugees, along with an even greater degree hostility toward them, was displayed in the migration crisis on the Polish-Belarusian border in Autumn 2021, caused by the actions of the Belarusian regime, with Russian support. The measures that Poland took in 2021 to repel refugees back to the Belarusian side of the border resulted in violations of the Geneva Convention on the Status of Refugees, the EU Charter of Fundamental Rights, the European Convention for the Protection of Human Rights and Fundamental Freedoms, which prohibits the collective expulsion of foreigners, and provisions of the Polish Constitution itself. In addition, Poland did not take in any refugees from Ukraine in the 8 years of the war in Ukraine since its beginning in 2014. The refusal of refugee applications to Ukrainian citizens was justified by the fact that not all of Ukraine's territory was threatened by hostilities, and they could therefore find safe refuge within their own country.

The situation changed on February 24^*th*^, 2022, when the entire territory of Ukraine was attacked by Russia. This caused a refugee movement to which Poland opened its borders.

No war or armed conflict in the 21^*st*^ century has yet provoked such a large migration to Poland as its main destination. As per the recent data available on the Ukraine Refugee Situation page of the United Nation's Operational Data Portal, a total of nearly 10 million border crossings from Ukraine to other countries have taken place since the February 24^*th*^. In the same period, there were nearly 3.7 million border crossings from other countries to Ukraine. Nearly half of all border crossings from Ukraine occurred on the Ukrainian-Polish border, as did nearly half of all border crossings into Ukraine.

Poland not only opened its borders but also has made a special law, that grants refugees from Ukraine access to the Polish labor market, health care and social assistance (Ustawa z dnia 12 marca, 2022). However, at this point, these state actions were not enough. The refugees needed housing, food, clothing, information and so on. Therefore, the grass-root host society of Poland has shown solidarity with the refugees and mobilized to help them.

The contrast of Poland‘s reaction on the Belarusian and Ukrainian borders indicates double standards. Helping and solidarity by the same activists on the Belarusian border was “criminalized,” while on another it was viewed very positively. Why is there such a difference? Why is the response of Polish society and authorities so diametrically opposed to Ukraine and the Polish-Belarusian border? The main explanation is that the Russian-Ukrainian war is understandable and is a threat to Poles, so they can understand the situation of Ukrainians.

But this is not the only answer. After all, there are reports of different treatment of refugees from Ukraine, for example, of Roma origin. Roma from Ukraine constitute a particular group of refugees due to their experience of discrimination in Ukraine and subsequently in Poland. This discrimination stems from a number of deeper cultural-historical issues which are resulting in problems that non-Roma refugees from Ukraine do not encounter in most cases. These include both verbal and non-verbal acts of discrimination and/or social and cultural exclusion from resources available to refugees: housing, jobs, information, transport, material resources, and psychological, legal and educational support (Mirga-Wójtowicz et al., 2022).

This shows that another reason why Poland treats refugees from both borders differently is prejudice. It's easy for Poles to find empathy and understanding for people who are close to them culturally, religiously, or even close in terms of appearance, but more difficult toward people they do not understand, do not understand what they say, do not understand what they believe. And they look different (Chrzczonowicz, 2022).

In this crisis, the psychological capital of the refugees as well as of the host society and its resources are very important, yet of primary importance is the help that war refugees need, in relation to the capabilities of the host country and its society. This is what the authors of this paper seek to investigate.

In this article short-term is used to describe things that will last for a short time, or things that will have an effect soon rather than in the distant future. Short terms needs and help are connected with emotional help, needed immediately, refers to basic human needs (in reference to Maslow's pyramid of needs). Something that is long-term has continued for a long time or will continue for a long time in the future. Long-term needs will be required in a long perspective of time, not immediately.

The study is limited and presents which short-term and long-term assistance needs by refugees of Ukrainian nationality.

This article is an attempt to diagnose the situation and share reflections from the field. Part of the added value of this article lies in the inclusion of the perspective of a researcher with a Ukrainian background, who works at the Ukrainian House in Warsaw and is an engaged observer of the diagnosed situation. The study we propound is exploratory and could serve as a good basis for a comprehensive study on the subject.

This article consists of the following parts: information about contextual data, an indication of the theoretical approach, statement of the research question, discussion of the research methods used, description of the results of the study and the drawing of conclusions from them with an indication of areas for future research.

## 2. Contextual data and studies

Before we present the sample of our exploratory study, we would like to discuss the population of Ukrainian refugees in Poland in administrative statistics and in two other studies conducted at a similar time in Poland as the one conducted by the authors of this article. According to information on the registration process for the Polish National Registration System PESEL, as of 15 May 2022, the number of registered Ukrainian refugees was as high as 1.1 million, with a very specific, feminized demographic structure (see Table 1). Among the registered persons, over 47% we children and youths (people up to age 18), 42% were females of working age, and almost 7% were elderly persons (retirement age, defined as 60+ for females and 65+ for males). The largest numbers of registrations took place in the biggest Polish agglomerations in the Mazovia (20%), Silesia (10%) and Lower Silesia (10%) regions (Duszczyk and Kaczmarczyk, 2022).

**Table 1:** Demographic data of general population of war refugees from Ukraine who registered for a PESEL number in Poland.

	**Number of war refugees**	**% of total**
Children (aged 0–18)	519,567	47.35%
Working age	503,071	45.85%
Female	460,361	41.96%
Male	42,710	3.89%
Retirement age	74,579	6.80%
Female	63,878	5.82%
Male	10,701	0.98%
Total	1,097,217	100%

Source: Duszczyk and Kaczmarczyk (2022), based on the PESEL register, data as of 15 May 2022.

In a survey conducted among refugees from Ukraine by the Interdisciplinary Research Laboratory regarding the war in Ukraine (*n* = 737) (76%) had university degrees, including master's and higher (64%). About three 52% of them material conditions. Most respondents lived in cities (91%), and most of them came from central Ukraine (46%). Forty-one percent of war refugees staying in Poland wanted to return to Ukraine as soon as the war ends, while 17% of them planned to stay in Poland permanently. Poland is the main country of migration chosen by people escaping from Ukraine. When asked about the reasons for this decision, the participants most often replied that they had family or friends in Poland (44%) or that Poland is a culturally similar country (42%). Other reasons included that it is possible to get from Ukraine to Poland rapidly (25%), and that Poland is relatively close to their home in Ukraine (24%). For some respondents, an important factor was also the aid provided to Ukrainians by Poles (20%) and the fact that Poland is a member of NATO, and they can feel safe here (15%). Only 6% of the moving to Poland before the outbreak of war in February (Długosz, 2022).

Regarding the professional situation of Ukrainian refugees in Poland, the results of a survey conducted by EWL Group (*n* = 400)^1^ showed that a significant proportion of the respondents before the fled to Poland worked in the services and trade sector (27%) and in education sectors (15%). Many of the surveyed refugees were highly qualified professionals (17%). Only 9% of the respondents declared that they had a good or very good knowledge of the Polish language, and as many as four-fifths of the refugees had never worked in Poland before. At the same time, most respondents (63%) wanted to work during their stay in Poland. At the time of the survey, one in five respondents declared that they were living on their own financial resources (20%) (Raport EWL “Uchodzcy z Ukrainy w Polsce”, 2022).

Despite the presence of 1.1 million war refugees from Ukraine in Poland as of October 1, only 58 Ukrainians citizens had refugee status in Poland. The reason is that Poland, with the opening of its borders to the mass migration of refugees under a special law, has provided them with a different formal-legal status (Ustawa z dnia 12 marca, 2022).

## 3. Theory: Aid attitudes

In order to provide aid, in addition to collective resources such as social solidarity, people also require individual resources to aid others.

Two of the most accurate concepts with regard to collective resources required for aid are social solidarity and aid attitudes. According to Durkheim (1933), social solidarity is the synergy between individuals in a society that aims for social order and stability. It underlines the interdependence and interplay between people in a society, which makes them feel that they can better the lives of others. The theory of social solidarity by Durkheim can be reflected in reducing social distance and social exclusion (cf. Mishra and Rath, 2020). Solidarity is the binding force that cements individuals based on normative obligations that facilitate collective action and social order (Hechter, 2018). Solidarity is meant as opposite to the values of individualism, social and market competition, purely instrumental rationality and its main meanings are unselfishness and a will to act in the interest of other people (Komter, 2001, 2005). Social solidarity not only involves common responsibility for the well being of members of the community (Paskov, 2012), but also emphasizes taking care of the needs and interests of underprivileged and vulnerable people.

In response to the Sustainable Development Goals, the Focus 2030 project was created. Focus 2030 supports international development actors working to promote effective and transparent public policies to achieve equality, poverty reduction and the UN Sustainable Development Goals by 2030. The aim of the Sustainable Development Goals, a series of 17 objectives fixed by the United Nations and adopted by 193 countries, is to create the guarantee of a better life for everyone, and a basis for a more stable, environmentally friendly, and equal world by 2030. Focus 2030's aim is to help keep international development on the agenda. One of the projects realized by Focus 2030's is the Aid Attitudes Tracker, a survey conducted in France, the United Kingdom, Germany and the United States. Aid attitudes can be understood as opinions, behaviors and levels of individual engagement (cf. Aid Attitudes Tracker^2^). We mention this tool with high hopes of expanding the countries that could be analyzed to include Poland or Ukraine. We think it deserves attention.

Let us consider the process of aid provision from a psychological perspective. A supportive attitude consists of three components: emotional, cognitive and behavioral (Breckler, 1984). The emotional component is what one feels toward another person, the cognitive component are one's thoughts and beliefs toward another person, and the behavioral component concerns the actual acts of providing aid. Attitudes are relatively constant assessments—positive or negative—of people, objects, and concepts (Eagly and Chaiken, 1993). Usually, people tend to think of themselves in a positive way, as decent, competent, sympathetic and honorable (Aronson et al., 2007).

Bearing the above characteristics in mind, a person's decision to aid or not is a complex process. Latane and Darley (1970) proposed a five-step decision model of helping, during each of which people can decide to do nothing (do not help): (1) notice the event (or in a hurry and not notice), (2) interpret the situation as an emergency (or assume that as others are not acting, it is not an emergency), (3) assume responsibility (or assume that others will do this), (4) know what to do (or not have the skills necessary to help), and (5) decide to help (or worry about danger, legislation, embarrassment, etc.).

An important human resource in the helping process is Psychological Capital. Psychological Capital is constructed of four main psychological resources: self-efficacy, hope, optimism and resilience (cf. Newman et al., 2014) which correspond to (a) the ability to face challenges (self-efficacy); (b) having positive attitudes toward present and future success (optimism); (c) the ability to adjust one's path to success (hope), and(d) the ability to recover and move on when faced with difficulties (resilience) (Luthans, 2002).

The most important theoretical models include the reasons of pro-social behavior are: the theory of social exchange (Thibaut, 1959; Homans, 1961), the norm of reciprocity (Aronson et al., 2007), the theory of mutual altruism (Trivers, 1971) and arousal-balance model (Piliavin et al., 1981). The theory of social exchange concerns searching for the motives of pro-social behavior in the pursuit of maximizing profits and minimizing costs. On the other hand, the norm of reciprocity is the assumption that others will treat us in the same way that we treat them. The theory of mutual altruism says that helping other members of one's own species also has benefits for the helper, as long as it is reciprocated. The theory of mutual altruism explains the phenomenon of helpfulness. Helping others increases one's resources by borrowing” from others. Thus, arousal-balance model, is about reducing or eliminating the tension that arises in a person as a result of watching someone else suffer (Piliavin et al., 1981).

After February 24^*th*^, a great “aid movement” arose in Poland. Almost every person prepared gifts—clothes, chemicals, toys and more—in order to help. The aim of our article is to try to capture both perspectives—that of people in need, and that of those who offer aid in the form of resources. Against the backdrop of the conceptual approach—an interplay of solidarity, aid attitudes and psychological capital—we formulate the following research questions:

1. Who are the people who need help, and who are people who offer it?
2. What do refugees need in the short term, and what do aid providers offer them?
3. What do refugees need in the long term, and what do aid providers offer them?

## 4. Methodology

Our exploratory, small-scale survey started 3 weeks after the Russian invasion of Ukraine on the February 24^*th*^ 2022 and was conducted in close cooperation between the NGO Ukrainian House in Warsaw and Center for Research on Social Change and Human Mobility. The study was designed in the first weeks after the start of the war. The list of possible forms of help was designed based on current assistance activities in Poland, as well as based on individual interviews with refugees who applied for help to the Ukrainian House in Warsaw. The survey was launched on March 8^*th*^ and data was collected until June of the same year. The survey measured psychological capital and forms of short-term and long-term help in two perspectives: people who offered help and the aid needs of refugees.

Data collection was carried out both on-line and on-site data at the premises of our partner NGO. We conducted the survey in three languages: Ukrainian, Polish, and English. We used a multi-channel recruitment approach, mostly through Facebook page and activities on the ground of our partner. A total of 218 people participated in the study. Most of them were women (*n* = 194; 89.0%) with higher education (1st, 2nd or 3rd level of education; *n* = 176; 80.8%). Over 65% (*n* = 142; 65.1%) of the respondents had children. Most (*n* = 168; 77.1%) had not experience migration for a period longer than 12 months before the war. More than half of the respondents had Ukrainian citizenship (*n* = 136; 62.4%) and were born in Ukraine (*n* = 133; 61.0%). The rest of the people were of Polish nationality. At the time of the study, most people were in Poland (*n* = 207; 96.3%).

Almost half of the respondents offered aid in connection with the war (*n* = 97; 44.5%); 84 people (38.5%) needed aid in connection with the war; 17 people (7.8%) both needed and offered aid (see Table 2). On the other hand, 20 people described themselves as observers—they neither needed nor offered aid to refugees. The research also recorded to locations were aid was provided / received. Most of the respondents needed / offered help in Poland (*n* = 165; 75.7%), on the Internet (*n* = 41; 18.8%) and in both Poland and Ukraine (*n* = 37; 17.0%). Among those who need help, 82 people filled in the questionnaire in Ukrainian, two people in Polish. Among those who offer help, 64 filled in the tool in Polish and 33 in Ukrainian. Information about citizenship, country of origin or country of residence of those who need and offer help are in Table 4.

**Table 2:** Descriptive statistics of selected qualitative variables.

**Variable**	**Level**	***n***	***%***
Perspective	I need help in connection with the war	84	38.5
	I offer aid in connection with the war	97	44.5
	None of the above	20	9.2
	Both of the above	17	7.8
Gender	Woman	194	89.0
	Man	23	10.6
Have children	Yes	142	65.1
	No	76	34.9
Citizenship	UA	136	64.8
	PL	74	35.2
Country of origin	UA	133	63.3
	PL	77	36.7

In our study, we evaluated Psychological Capital (PsyCap) using the Polish and Ukrainian translation of the Compound Psychological Capital Scale CPC-12 (Lorenz et al., 2016). This scale consists of 12 self-evaluating statements rated on a 6-point Likert scale (ranging from 1 = strongly disagree to 6 = strongly agree). The translation process followed the guidelines for the translation and adaptation of psychological instruments (Stajkovic and Luthans, 1998). Two persons, first fluent in English-Polish and second in English–Ukrainian, carried out parallel translations of the instrument. The inconsistencies between the independent translations were settled by a PsyCap expert.

We divided the forms of short-term and long-term aid into the following categories: material, psychological, humanitarian, organizational and professional. In total, there were 22 possible forms of needed and offered aid on the list.

## 5. Findings

In the first step (see Table 3), we determined who are people who need and who are people who offer help?

**Table 3:** List of possible forms of needed/offered aid and help needs of refugees.

**Kinds of help/aid**	**Category**
Money	Material
Flat (place to sleep)	Humanitarian
Food	Humanitarian
Clothes	Humanitarian
Hygiene products	Humanitarian
Support from a psychologist	Psychological
Support groups	Psychological
Legal help	Organizational
Help in finding a job	Professional
Career counseling	Professional
Help in finding an apartment for rent	Organizational
Assistance in recognizing or confirming education obtained abroad	Organizational
Support in completing formalities in offices	Organizational
Technological assistance	Organizational
Symbolic help (e.g., UA flag on social media profile)	Psychological
Blood donation	Humanitarian
Volunteering (participation)	Organizational
Volunteering (aid organization)	Organizational
Helping Ukrainian soldiers	Humanitarian
Free passenger transport	Organizational
Free transport of goods	Organizational
Learning Polish	Organizational

**Table 4:** Descriptive statistics of people in need of help and people providing aid.

**Variable**	**Level**	**Need help (*****n*** = **84)**		**Offer help (*****n*** = **97)**	
		***n***	**%**	***n***	**%**
Gender	Woman	79	95.2	82	84.5
	Man	4	4.8	15	15.5
Have children	Yes	60	71.4	60	61.9
	No	24	28.6	37	38.1
Citizenship	UA	81	98.8	26	28.6
	PL	1	1.2	65	71.4
Country of birth	UA	76	97.4	29	30.2
	PL	2	2.6	67	69.8
Country of residence at the time of the survey	UA	6	7.1	1	1.0
	PL	77	91.7	95	97.9

The people in need of help are mainly women (94.0%) who have children (71.4%). They were born in Ukraine (97.4%), have Ukrainian citizenship (99.8%), and were living in Poland at the time of the study (91.7%). The people offering aid are also mostly women (84.5%) who also have children, though to a lesser degree (61.9%), with Polish (71.4%) or Ukrainian (28.6%) citizenship, mostly born in Poland (69.8%). Almost all lived in Poland at the time of the study (97.9%).

We compared people who offered aid by gender and discovered two interesting results. First, most of the men who helped had children (*n* = 11; 73%) compared to women (*n* = 49, 59%). Secondly, mainly people without migration experience helped, although the percentage of women was lower (*n* = 53; 64%) than men (*n* = 11; 73%). Other characteristics of age, education or company size are almost the same.

We then investigated the psychological capital, psycho-physical condition and relationships with family and friends of people who need and people who provide aid. For this purpose, tests were performed for independent groups. This revealed that people offering help have a significantly higher psychological capital (except for optimism, where no statistically significant differences were observed). Their psycho-emotional condition is better, as are their relationships with family and friends (see Table 5).

**Table 5:** Descriptive statistics of people needing help and offering aid.

	**Need help**		**Offer help**		***t***	**df**	***p*-Value**
	***M***	***SD***	***M***	**SD**			
Hope	10.73	3.07	13.44	2.82	−6.210	179	< 0.001
Self-efficacy	11.51	3.28	13.76	2.91	−4.897	179	< 0.001
Resilience	11.75	3.75	13.88	3.22	−4.107	179	< 0.001
Optimism	13.88	3.87	14.69	3.37	−1.505	179	n.i.
Psycho-emotional condition	3.48	1.46	4.62	1.28	−5.612	179	< 0.001
Physical condition	4.33	1.39	4.60	1.41	−1.266	179	n.i.
Relationships with family	5.20	1.55	5.77	1.30	−2.698	179	0.008
Relationships with friends	5.08	1.61	5.76	1.21	−3.242	179	0.001

n.i., not important/no significant difference between groups.

In the next step, we compared what refugees from Ukraine need and what forms of short-term and long-term assistance they receive in Poland.

Our analysis of short-term aid showed that refugees most frequently declared a need for help in learning Polish, material assistance, money and help in finding a job. The most frequently offered forms of aid were money, clothes, and hygiene products. Analyzing the results in terms of the gaps or mismatches between the needs and the help offered, we see significant discrepancies. Shortages, i.e., needs exceeding the offered support, were observed for learning Polish, help in finding a job and psychological support, as most refugees had experienced trauma during the Russian invasion of Ukraine (see Table 6). In the table, the top three most needed and most offered forms of aid are indicated between parentheses after the relevant values, with the number in parentheses indicating the ranking of the needed/provided aid.

**Table 6:** Forms of short-term aid from the perspective of the needs of refugees, the aid provided by supporters, and the difference between them.

**Forms of short-term help/support**	**Needed help [A]** ** (*n* = 84)**	**Provided aid [B]** ** (*n* = 97)**	**Difference** ** [A – B]**
Learning Polish	54 (1)	12	42
Help in finding a job	43 (3)	16	27
Psychological—support from a psychologist	31	12	19
Career counseling	22	5	17
Legal	19	6	13
Assistance in recognizing or confirming education obtained abroad	13	3	10
Childcare	12	2	10
Assistance in finding an apartment for rent	29	20	9
Psychological—support groups	11	4	7
Support in completing formalities in offices	15	13	2
Free transport of people	8	7	1
Free transport of goods	4	5	−1
Blood donation	1	4	−3
Technological assistance	3	7	−4
Helping Ukrainian soldiers or the Ukrainian Army	12	17	−5
Material—money	50 (2)	59 (1)	−9
Humanitarian—an apartment (a place to sleep)	12	25	−13
Voluntary work (aid organization)	2	20	−18
Humanitarian—food	24	43	−19
Humanitarian—hygiene products	19	45 (3)	−26
Symbolic help (e.g., Ukrainian flag on social media profile)	2	28	−26
Humanitarian—clothes	16	47 (2)	−31
Volunteering (participation)	11	50	−39

Analyzing the long-term needs for and provided aid, we again, see many discrepancies. The most common long-term needs of refugees include material help, learning Polish, career counseling and help in finding a job. Meanwhile, the help that is offered to refugees in the long term is money, food, clothing, and volunteering (see ranked forms in parentheses in Table 6). The forms of assistance subject to the greatest shortages concern learning Polish, career counseling and help in finding a job. We found the greatest excesses of offered assistance in giving clothes, symbolic help (e.g., UA flag on social media profile) and volunteering (participation; see Table 7). Again, the numbers in parentheses after the values indicate the ranking of the three most needed and most offered forms of aid.

**Table 7:** Forms of long-term aid from the perspective of the needs of refugees, the aid provided by supporters, and the difference between them.

**Forms of long-term help/support**	**Needed help [A]** ** (*n* = 84)**	**Provided aid [B]** ** (*n* = 97)**	**Difference** ** [A – B]**
Learning Polish	44 (2)	4	40
Career counseling	28	4	24
Help in finding a job	38 (3)	18	20
Assistance in recognizing or confirming education obtained abroad	17	3	14
Psychological—support from a psychologist	21	9	12
Assistance in finding an apartment for rent	24	13	11
Childcare	12	3	9
Legal	14	6	8
Psychological—support groups	12	6	6
Helping Ukrainian soldiers or the Ukrainian Army	16	11	5
Free transport of people	8	4	4
Material—money	45 (1)	45 (1)	0
Support in completing formalities in offices	15	15	0
Humanitarian—an apartment (a place to sleep)	9	12	−3
Free transport of goods	2	5	−3
Technological assistance	1	5	−4
Blood donation	2	6	−4
Humanitarian—food	20	35 (3)	−15
Humanitarian—hygiene products	17	32	−15
Voluntary work (aid organization)	2	18	−16
Humanitarian—clothes	14	32	−18
Symbolic help (e.g., UA flag on social media profile)	1	23	−22
Volunteering (participation)	10	38 (2)	−28

## 6. Conclusions and discussion

The study shows that among our respondents, the people in need of help and the people who offered aid were primarily women, most of whom had children. This follows from the fact that refugee women are primarily women with children. Considering that the people offering aid in the host society were also primarily women with children, this may be indicative of an attitude empathy and solidarity among this group.

Respondents who offered aid had higher psychological capital, which may, among other things, contribute to their willingness to help refugees. Psychological help, is an important need for refugees, many of whom have experienced war trauma. People offering aid have a significantly higher psychological capital (except for optimism, where no statistically significant differences were observed) than those who need help. Which means they have more internal resources to help others.

Our study found numerous gaps between the needed and offered short-term and long-term aid as indicated by the respondents. First and foremost, this is because initial aid attitudes were primarily driven by emotions. According to the arousal-balance model, the sight of someone else's misfortune arouses unpleasant emotional arousal in the observer, and the observer will try to defuse it in the quickest and simplest way possible (Piliavin et al., 1981). In other words, the emotional drive and desire to help overpowered the rational deliberation of what form this aid should look take and what needs it should meet. In addition, no aid management system was in place or put in place, meaning that no information was available regarding best practices for aid and what forms of aid were needed in the first place. Polish respondents have therefore primarily offered aid in the form of hygiene products, food, clothing, and money for collections. This aid was at times chaotic, excessive, and much of it ended up in trash cans (especially food). The aid provided by respondents was primarily these forms of aid, of a of ore symbolic nature, while refugee respondents primarily indicated a need for different kind of aid, namely support in stabilizing them and precarious living situation. To be more specific, refugee respondents first and foremost indicated a need for assistance relating to opportunities to support themselves and their families: assistance in finding a job and learning the Polish language. Due to the sheer size of the group of refugees and the reason for their migration, an entire cross-section of Ukrainian society resides in Poland, from ordinary workers to highly skilled professionals running their own businesses. To find their way on the Polish labor market, they need support in the form of courses, training, career counseling, recognition of education and work experience and, most importantly, learning the Polish language. The range of courses offered is still very limited and does not meet the extensive demand.

A major barrier to the state's provision of long-term support to refugees, which is proving difficult to overcome, is the structural weakness of the public service system, especially as concerns medical care and housing. The fact that access to these services is already difficult for the Polish public means that it is essentially not possible to provide real support to more than a million refugees. From the perspective of one of the authors of this article, as a leader of an NGO that aid refugees, housing remains an important need for refugees, the importance of which increases as the autumn and winter seasons approach the host society's aid fatigue grows. Poles are no longer willing to offer temporary housing in their homes, and the rental housing markets of major cities, where refugees are primarily located, are unable to respond to the high demand. The high demand in the housing market combined with fast-moving inflation is causing a continues rise in prices to a level that mothers with children to afford, considering their financial capacity, simply cannot afford.

The expression of solidarity from the Polish people that we discussed in this article is reflected in the understanding that the state was initially incapable of a rapid respondsee to the crisis. This was due to a lack of experience and an absence of strategies for migration and integration policies. Polish society and NGOs therefore stood up to take the first “blow” of the refugee influx by providing short-term assistance, thereby giving the government time to plan out the long-term support. However, the state has not taken any responsibility for assistance and integration upon itself. As a result, the mismatch between the needed and provided aid from the long-term perspective causes adverse reactions in society, resulting in a reluctance to provide aid due to the perceived ungratefulness on the part of refugees. Furthermore, there is a widespread lack of knowledge about best practices for aid provision. The lack of real long-term support is causing some of refugees, having exhausted their financial resources, to seek help in other European countries or return home, where they remain in danger.

The results of this study indicates that there is a need to study psychological capital among people who offer aid and need help. This will help to understand the importance of the psychological capital of refugees and the host society in dealing with the present crisis. The identified problems require further thorough research to investigate, on a larger sample of respondents, the matching of needs and assistance. Such a follow-up study could be enriched with the perspective of NGO staff working on behalf of migrants and refugees, as well as state and local government employees. The results of such a study could be the basis for recommendations to various actors as to how the situation may be improved.

## Data availability statement

The original contributions presented in the study are included in the article/supplementary material, further inquiries can be directed to the corresponding author.

## Ethics statement

Ethical review and approval was not required for the study on human participants in accordance with the local legislation and institutional requirements. The patients/participants provided their written informed consent to participate in this study.

## Author contributions

All authors listed have made a substantial, direct, and intellectual contribution to the work and approved it for publication.

## Footnotes

^1^The survey was carried out using direct individual interviews with the use of tablets (CAPI F2F), from March 23 to 3 April in two big cities in Poland—Warsaw and Cracow.

^2^ https://focus2030.org/The-Aid-Attitudes-Tracker-project
